# Supplementary figures and images for: Integrin α3β1 in hair bulge stem cells modulates CCN2 expression and promotes skin tumorigenesis
Source: Life Sci Alliance. 2020 May 18;3(7):e202000645. doi: 10.26508/lsa.202000645 (PMC7240742; doi:10.26508/lsa.202000645)

integrin  $\alpha 3$   
light chain

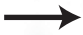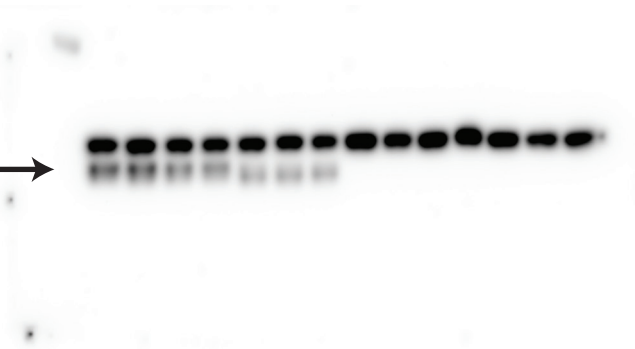

CCN2

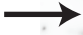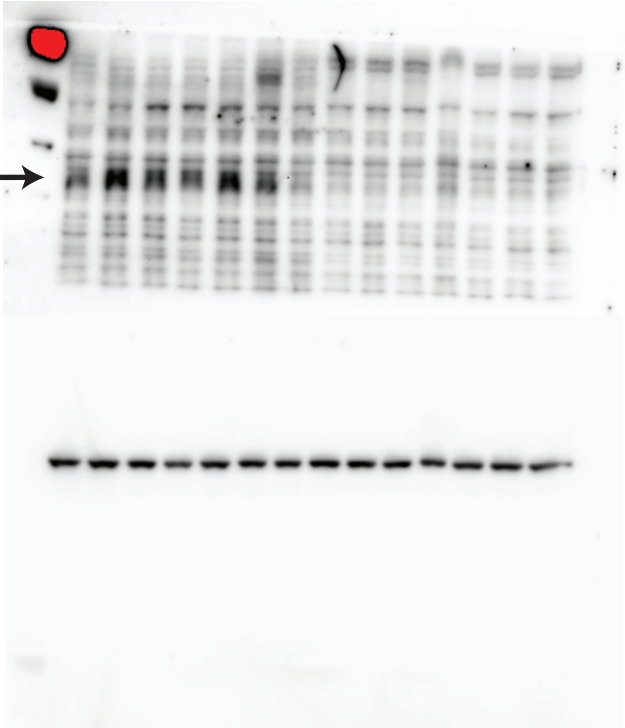

GAPDH

Supplement: Supplementary file 4 [file LSA-2020-00645_SdataF7.pdf]

integrin  $\alpha 3$   
precursor

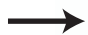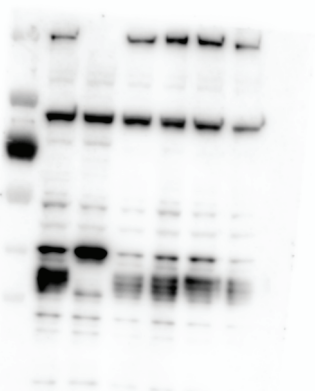

integrin  $\alpha 3$   
light chain

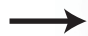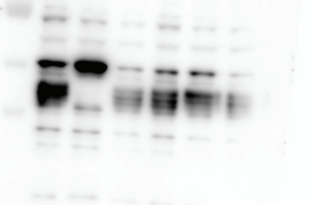

CCN2

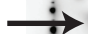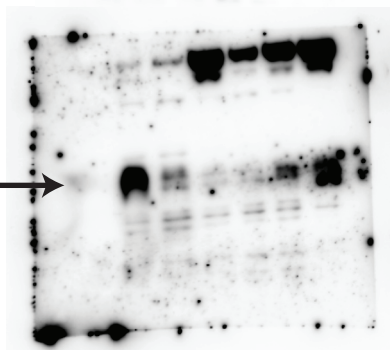

GAPDH

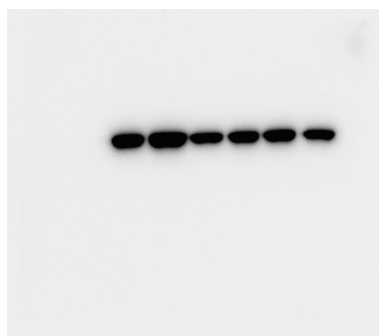

Supplement: Supplementary file 5 [file LSA-2020-00645_SdataF8.pdf]
